# Wireless power transfer system rigid to tissue characteristics using metamaterial inspired geometry for biomedical implant applications

**DOI:** 10.1038/s41598-021-84333-3

**Published:** 2021-03-12

**Authors:** Ramesh K. Pokharel, Adel Barakat, Shimaa Alshhawy, Kuniaki Yoshitomi, Costas Sarris

**Affiliations:** 1 Graduate School of Information Science and Electrical Engineering, Kyushu University, Nishi-ku, Fukuoka, 819-0395 Japan; 2 The Edward S. Rogers Sr. Department of Electrical and Computer Engineering, University of Toronto, Toronto, ON M5S 3G4 Canada

**Keywords:** Biomedical engineering, Electrical and electronic engineering

## Abstract

Conventional resonant inductive coupling wireless power transfer (WPT) systems encounter performance degradation while energizing biomedical implants. This degradation results from the dielectric and conductive characteristics of the tissue, which cause increased radiation and conduction losses, respectively. Moreover, the proximity of a resonator to the high permittivity tissue causes a change in its operating frequency if misalignment occurs. In this report, we propose a metamaterial inspired geometry with near-zero permeability property to overcome these mentioned problems. This metamaterial inspired geometry is stacked split ring resonator metamaterial fed by a driving inductive loop and acts as a WPT transmitter for an in-tissue implanted WPT receiver. The presented demonstrations have confirmed that the proposed metamaterial inspired WPT system outperforms the conventional one. Also, the resonance frequency of the proposed metamaterial inspired TX is negligibly affected by the tissue characteristics, which is of great interest from the design and operation prospects. Furthermore, the proposed WPT system can be used with more than twice the input power of the conventional one while complying with the safety regulations of electromagnetic waves exposure.

## Introduction

Biomedical implants can monitor the vital signs of the human body^1–3^. A fundamental requirement of such an implant is the size compactness to ensure the comfort of the treated person. Therefore, instead of bulky batteries, the essential energy for the operation of these biomedical implants can be supplied externally by exploiting wireless power transfer (WPT) technology^2–23^. Still, the use of WPT technology adds some constraints such as high transfer efficiency to minimize the exposure time to the electromagnetic (EM) waves^10^. Also, a low specific absorption rate (SAR) is crucial to assure body safety from these EM waves^1–3,8–12,23^.

Sensitivity to misalignment effects^5,6,24,25^ is a major issue in WPT systems including the inductively coupled one. Misalignment is caused by the change of vertical separation between transmitter (TX) and receiver (RX), lateral shift between their axes, angular change from their axes, or a combination of these types. Misalignment results in a change of the coupling between TX and RX. Therefore, the efficiency of the WPT system reduces due to mismatch loss or reduction in obtainable efficiency^26^. Additional problems arise when the WPT system is used for biomedical implant applications. First, propagation into a lossy medium results in degradation of the efficiency^1–3,8–12^. Second, the conventional methods in optimizing the coupled quality factor^26^ may result in a TX that has its resonant frequency dependent on the tissue characteristics. Consequently, this TX resonant frequency may change when misalignment occurs resulting in severe degradation in the efficiency due to frequency mismatch between the TX and RX.

**Figure 1:**
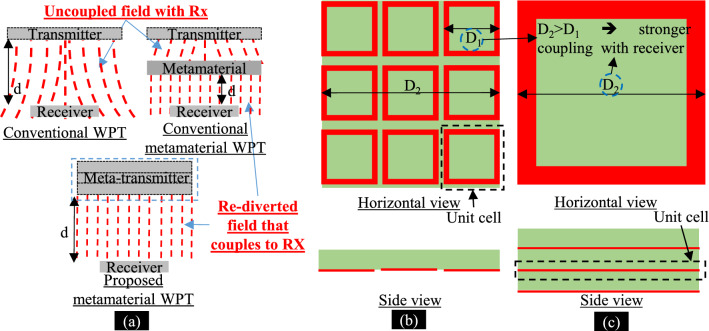
Near-Zero metamaterial. **(a)** Conventional and proposed WPT systems. **(b)** Conventional planar metamaterial in 3 × 3 array configuration. **(c)** Proposed metamaterial inspired geometry in co-axial configuration.

Recent advancements in WPT systems have focused on the deployment of meta-surface^13–15^, or metamaterial^17–21^. Such meta-surfaces or -materials have negative or near-zero effective permeability or permittivity characteristics that help to improve the coupling between the TX and RX to enhance the performance of the WPT system. Younesiraad et al.^13^ proposed the equivalent surface impedance concept by meta-surface, which enabled an extended WPT range by proper selection of this impedance. The active routing-based meta-surface^14^ enabled the adaptive location of the receiver during operation. Lang and Sarris^15^ proposed a semidefinite-relaxation-based method to determine the optimum loading reactance of the meta-surface unit cells. The resulting meta-surface showed enhanced efficiency when utilized as a passive relay in a reference WPT system when compared to that WPT system without meta-surface^15^. In^17–21^, further intermediate meta-surface or metamaterials layers with near-zero, negative permeability, or both of them have been exploited to enhance the WPT systems efficiency. Still, current WPT systems based on meta-surface^13–15^, or metamaterial^17–21^ suffer from a reduction of the working distance, i.e. the distance between the receiver and the meta-surface itself as can be interpreted from Fig. 1a.

In this report, we propose a new category of metamaterial inspired geometry where the unit cells are stacked in a co-axial configuration. Then, we employ this stacked geometry as a WPT transmitter (Meta-TX) that transfers the power to an embedded WPT receiver in biological tissues. Hence, the working distance is kept as the original distance between the TX and RX of the WPT system. The proposed metamaterial inspired geometry has near-zero effective permeability and can divert more magnetic fields towards the receiver resulting in efficiency and misalignment performance enhancement.

## Results

### Magnetic field and dispersion characteristics of a small inductive loop

An inductive loop placed in the $xy$-plane and has its center at z = 0 is a magnetic dipole whose near-field ($\beta r\ll 1$) magnetic field is described as^27^

1 $$\left[\begin{array}{c}{H}_{r}\\ {H}_{\theta }\\ {H}_{\varphi }\end{array}\right]={H}_{0}\left[\begin{array}{c}2\mathrm{cos}\theta \\ \mathrm{sin}\theta \\ 0\end{array}\right]$$

where ($r$, $\theta$, $\varphi$) represents the spherical coordinates system, $\beta$ is the phase constant, and ${H}_{0}=IA/4\pi {r}^{3}$. Here, assuming a single-turn loop, $I$ and $A$ are the current in this inductive loop and its area, respectively. Equation (1), by applying a coordinate transformation, can be rewritten in the cylindrical coordinates system as

2 $$\left[\begin{array}{c}{H}_{\rho }\\ {H}_{\varphi }\\ {H}_{z}\end{array}\right]=\left[\begin{array}{ccc}\mathrm{sin}\theta & \mathrm{cos}\theta & 0\\ 0& 0& 1\\ \mathrm{cos}\theta & -\mathrm{sin}\theta & 0\end{array}\right]\times \left[\begin{array}{c}{H}_{r}\\ {H}_{\theta }\\ {H}_{\varphi }\end{array}\right]=\frac{{H}_{0}}{2}\left[\begin{array}{c}3\mathrm{sin}2\theta \\ 0\\ 1+3\mathrm{cos}2\theta \end{array}\right]$$

The component ${H}_{\rho }$ is parallel and cannot generate near-field coupling. Alternatively, the component ${H}_{z}$ is in the perpendicular direction and can achieve near-field coupling. Where ($\rho$, $\varphi$, $z$) represents the cylindrical coordinates system. Besides the two magnetic field components, the electric field has one component^27^, which is ${E}_{\varphi }.$ Hence, ${k}_{\varphi }$ has a zero value and the wave vector can be written as:

3 $$ \vec{\user2{k}} = k_{\rho } {\varvec{a}}_{{\varvec{\rho}}} + k_{z} {\varvec{a}}_{{\varvec{z}}} $$

where ${k}_{\rho }$, ${k}_{\varphi }$ and ${k}_{z}$ are the wavenumbers in $\rho$-, $\varphi$-, and $z$-directions. Assuming diagonal permittivity ($\varepsilon$) and permeability ($\mu$) and ${\varepsilon }_{\rho }={\varepsilon }_{\varphi }={\varepsilon }_{z}$ and ${\mu }_{\rho }\ne {\mu }_{\varphi }\ne {\mu }_{z}$. The wave equation can be written as^28^

4 $$\overrightarrow{{\varvec{k}}}\times \overrightarrow{{\varvec{k}}}\times \overrightarrow{{\varvec{H}}}+\frac{{\omega }^{2}}{{c}^{2}}\mu \varepsilon \overrightarrow{{\varvec{H}}}=0$$

where $\omega$ and $c$ are the frequency and the speed of light. Utilizing (2)-(3) in (4) results in

5 $$\left[\begin{array}{ccc}{{\omega }^{2}\mu }_{\rho }{\varepsilon }_{\varphi }/{c}^{2}-{k}_{z}^{2}& 0& {k}_{\rho }{k}_{\rho }\\ 0& {{\omega }^{2}\mu }_{\rho }{\varepsilon }_{\varphi }/{c}^{2}& 0\\ {k}_{z}{k}_{\rho }& 0& {{\omega }^{2}\mu }_{z}{\varepsilon }_{\varphi }/{c}^{2}-{k}_{\rho }^{2}\end{array}\right]\left[\begin{array}{c}{H}_{\rho }\\ 0\\ {H}_{z}\end{array}\right]=0$$

The determinant of the coefficient’s matrix in (5) must vanish for non-trivial solutions of $\overrightarrow{{\varvec{H}}}$ to exist^28^**.** Hence, the dispersion equation in the cylindrical coordinates system of this small inductive loop can be written as

6 $$\frac{{k}_{\rho }^{2}}{{\mu }_{z}}+\frac{{k}_{z}^{2}}{{\mu }_{\rho }}=\frac{{\omega }^{2}}{{c}^{2}}{\varepsilon }_{\varphi }$$

Utilizing $\left|{\mu }_{z}\right|<1$, $\left|{\mu }_{p}\right|\ge 1$, and $\left|{\varepsilon }_{\varphi }\right|\ge 1$ results in minimization of the variance of the magnetic field in $\rho$-direction, i.e. ${k}_{\rho }\to 0$. Hence, the magnetic field is enforced in the $z$-direction as can be interpreted from Fig. 1a.

### Proposed metamaterial inspired geometry

A metamaterial based on a split-ring ring resonator consists of a group of periodic sub-wavelength patches or loops in one-, two- or three-dimensional configurations^13–23^. Such metamaterials are decisive as they empower anisotropic material characteristics such as near-zero permeability whose importance has been described in the previous section. The use of a two-dimensional array metamaterial, as shown in Fig. 1b, can achieve the target near-zero permeability. Still, the amplitude of the resulting magnetic field may be small due to the limited area of each unit cell. Alternatively, we propose the use of stacked metamaterial inspired geometry co-axially aligned in the $z$-direction as shown in Fig. 1c. Hence, near-zero permeability, as well as the comparably stronger amplitude of the magnetic field, can be achieved concurrently.

**Figure 2:**
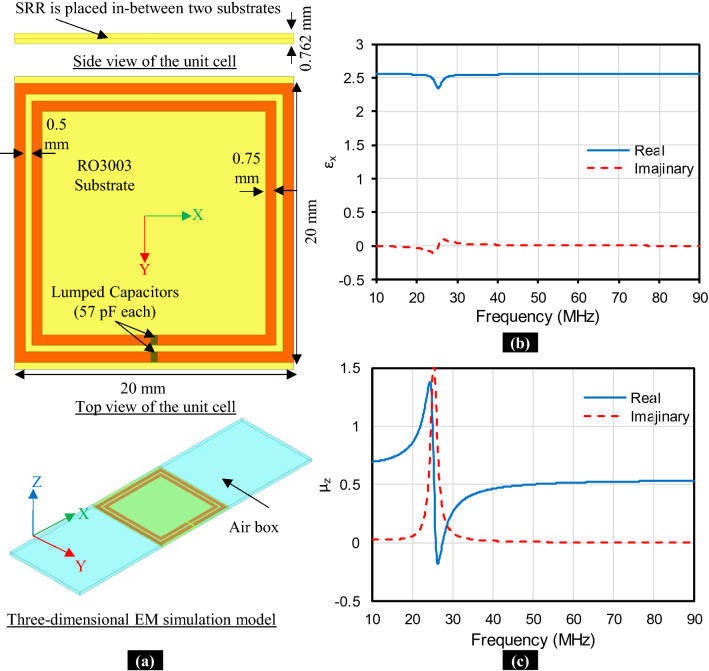
Proposed metamaterial inspired geometry. **(a)** Unit cell of SRR and EM simulation model. **(b)** Effective relative permittivity (ε_x_). **(c)** Effective relative permeability (μ_z_).

In Fig. 2a, the unit cell of metamaterial inspired geometry is shown as well as its EM simulation model. This unit cell consists of a split ring resonator (SRR). Each of the SRR rings is loaded by a compensating capacitor to control the frequency of operation. Wave ports are placed perpendicular to the Y-coordinate at the edge of the air-box. The proposed metamaterial inspired geometry has the unit cells of SRR stacked in the z-direction. So, the magnetic boundary conditions are placed perpendicular to the $z$-coordinate and just on the surface of the substrate. Finally, the electric boundary conditions are placed perpendicular to the x-coordinate at the edge of the air-box. We have extracted the effective material parameters, permittivity ($\varepsilon$), and permeability ($\mu$) from the complex scattering parameters as described in surface^29^. The extracted permittivity and permeability are shown in Fig. 2b,c, respectively. The extracted permeability shows near-zero characteristics in the z-direction at the frequency of interest, i.e. 50 MHz. Hence, the employment of this unit cell as the building block in a WPT system is expected to improve the performance of this system at 50 MHz.

### Proposed wireless power transfer system

**Figure 3:**
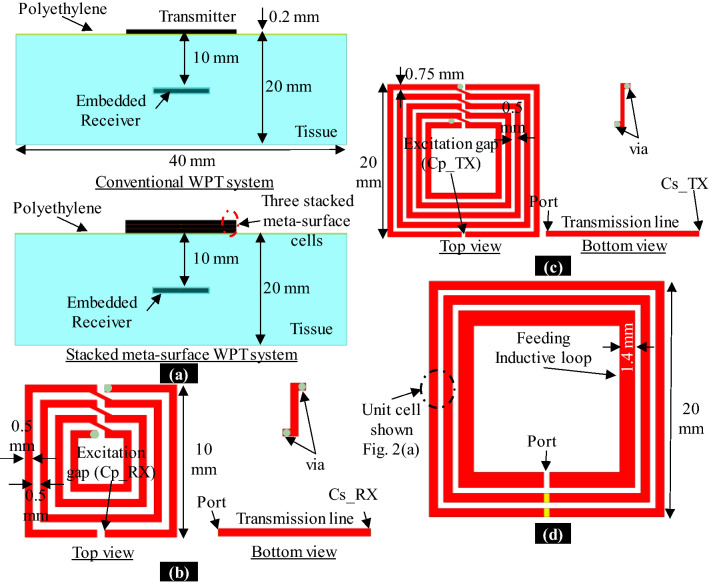
Conventional and proposed stacked metamaterial WPT systems in tissue. **(a)** Side view. **(b)** Layout of the RX in both cases. **(c)** Layout of conventional TX. **(d)** Layout of meta-TX.

In this section, we discuss the conventional resonant inductive coupling WPT system, as well as the proposed metamaterial inspired WPT system in the tissue environment. The side views of both systems are as shown in Fig. 3a. In both cases, the receiver is embedded inside a tissue (muscle type: dielectric constant = 77, conductivity = 0.68, and dielectric loss tangent = 3.16). These tissue material properties are considered based on^30^ at 50 MHz. The top and bottom layers of the RX are covered with a 0.2 mm thick polyethylene (dielectric constant = 2.25) to isolate the copper from the tissue. Also, the TX-tissue interface is covered by a 0.2 mm thick polyethylene for the same purpose.

#### System layout

The TX and RX are printed on the RO3003 substrate. The RX is a four-loop DGS resonator with the layout and dimensions shown in Fig. 3b. The receiving DGS resonator is loaded by a compensating capacitor ($Cp\_RX$) at its excitation gap and an admittance inversion capacitor ($Cs\_RX$) is connected between the transmission line and the ground. The optimized values of these capacitors are $Cp\_RX =69.5$ pF, $Cs\_RX =19$ pF in the case of conventional as well as proposed metamaterial inspired WPT systems. The conventional TX of the WPT system is a five-loop DGS resonator with the layout and dimensions as shown in Fig. 3c. This conventional TX is loaded by a compensating capacitor ($Cp\_TX =9$ pF) at its excitation gap and an admittance inversion capacitor ($Cs\_TX =9$ pF) is connected between the transmission line and the ground. Instead, the proposed SRR meta-TX consists of three co-axially aligned unit-cells and the upper cell is magnetically coupled to a feeding inductive loop with the dimensions noted in Fig. 3d.

#### Simulated performance

**Figure 4:**
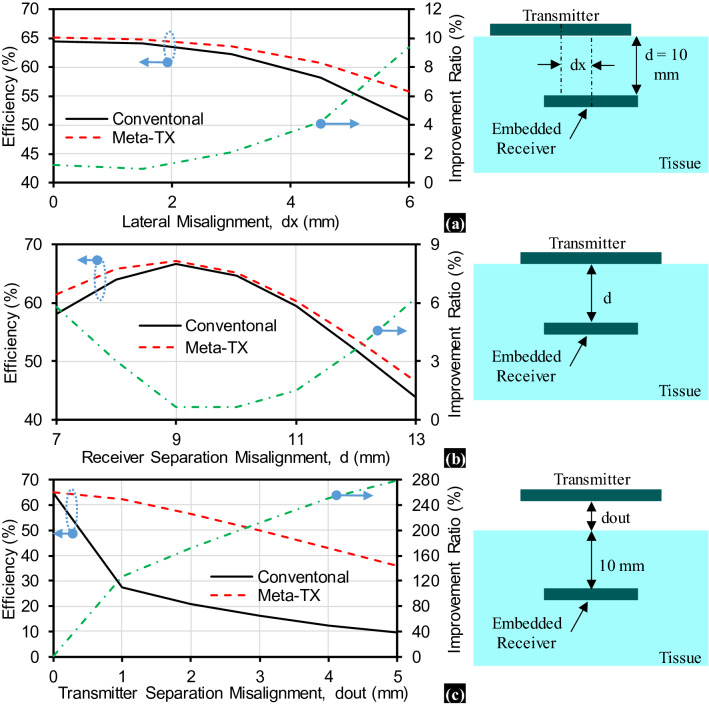
Simulated efficiency comparison between conventional and proposed metamaterial inspired WPT systems during misalignment **(a)** Lateral Misalignment. **(b)** Receiver Separation misalignment inside the tissue. **(c)** Transmitter separation misalignment from the tissue.

A major limitation of conventional WPT systems is their sensitivity to misalignment^5,6,24,25^. In Fig. 4, we study and compare the efficiency performance of the conventional and proposed metamaterial inspired WPT systems during lateral and separation misalignments. Efficiency is calculated as

7 $$ {\text{Efficiency}} = \left| {S_{21} } \right|^{2} $$

in which ${S}_{21}$ is the transmission coefficient at the frequency of interest, i.e. 50 MHz. The proposed metamaterial inspired WPT system has higher efficiency than that of the conventional WPT system when lateral misalignment occurs as shown in Fig. 4a. This represents an improvement ratio of about 10% when $\mathrm{dx}$ = 6 mm, which is 30% of the TX side length. The improvement ratio is calculated as

8 $$ {\text{Improvement ratio}} = \frac{{{\text{Efficiency}}_{{{\text{meta}} - {\text{surface}}}} - {\text{Efficiency}}_{{{\text{conventional}}}} }}{{{\text{Efficiency}}_{{{\text{conventional}}}} }} $$

**Figure 5:**
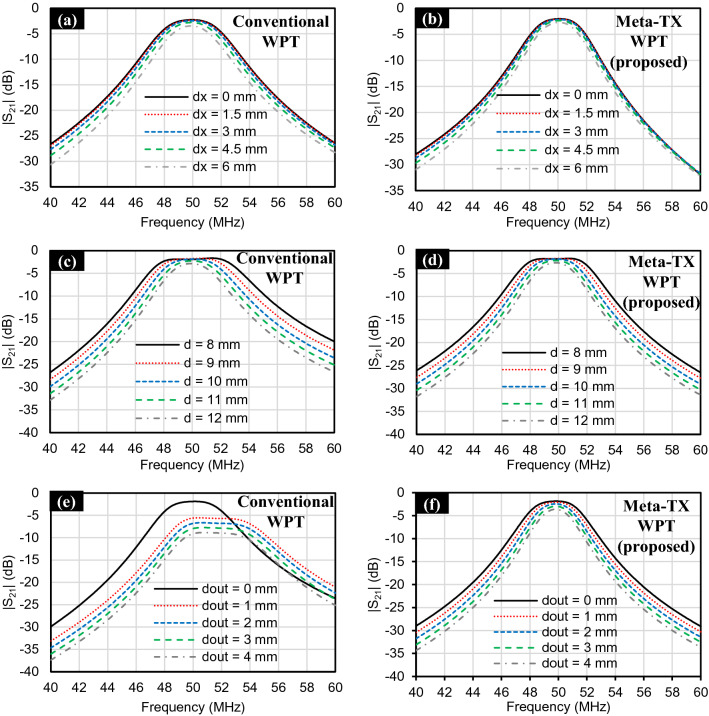
Simulated transmission coefficient (|S21|) of WPT systems during lateral misalignment: **(a)** Conventional. **(b)** Proposed; receiver separation misalignment inside the tissue. **(c)** Conventional. **(d)** Proposed; and transmitter separation misalignment from the tissue: **(e)** Conventional. **(f)** Proposed.

Efficiency degrades when lateral misalignment occurs because of the decrease of the transmission coefficient as shown in Fig. 5a,b. This decrease in the transmission coefficient results from the reduction of the magnetic coupling. Magnetic coupling reduction leads to both mismatch losses as well as reduction of the maximum obtainable efficiency^5,6,24,25^. The improvement in efficiency performance of the proposed metamaterial inspired WPT system during lateral misalignment is considered as proof of the magnetic field enforcement in the z-direction as discussed in the prior Sections. Furthermore, we test the efficiency performance during separation misalignment. Two cases of separation misalignment are considered:

- Changing of the implantation depth, $\mathrm{d}$, of the receiver as shown in Fig. 4b. We name it “RX separation misalignment”.
- Having a gap, $\mathrm{dout}$, between the transmitter and the tissue as shown in Fig. 4c. We name it “TX separation misalignment”.

The proposed metamaterial inspired WPT system shows better efficiency than that of the conventional WPT system when RX separation misalignment occurs. In Fig. 4b, the improvement ratio when utilizing the proposed metamaterial inspired WPT system is about 6% when $\mathrm{d}$ = 7 mm or 13 mm, which is ± 30% of the original implantation depth of the receiver, i.e. d = 10 mm. In Fig. 5c,d, we show the transmission coefficient that we have used to calculate the efficiency. In terms of TX separation misalignment, the proposed metamaterial inspired WPT system shows an outstanding improvement ratio of more than 200% when $\mathrm{dout}$ = 3 mm, which is 30% of the original separation between the TX and RX, i.e. d = 10 mm. The proposed metamaterial inspired WPT system maintains an efficiency of about 50% at $\mathrm{dout}$ = 3 mm while the conventional WPT system has only 16% efficiency. The reason behind this efficiency performance can be understood from the transmission coefficient results in Fig. 5e,f. The conventional WPT system suffers from a resonance change once the TX is separated from the tissue, which is not the case in the proposed metamaterial inspired WPT system. The change in the resonance, in the case of the conventional WPT system, has occurred because the TX consists of several loops connected in series and suffer from increased effective inductance due to the relatively large parasitic capacitance resulting from the dielectric characteristics of the tissue. Effective inductance is the parallel combination of the intrinsic inductance and the parasitic capacitance from the medium. On the contrary, the proposed metamaterial inspired WPT system consists of non-connected loops that mutually resonate at the desired frequency and each of them has a self-resonance that is not greatly affected by approaching the tissue. Also, the unit cell interfacing the tissue shields the other cells from the tissue. In summary, the proposed stacked SRR metamaterial inspired WPT system does not have a dependency on the tissue characteristics, which is an important issue for biomedical implants.

#### Fabrication and measurement results

**Figure 6:**
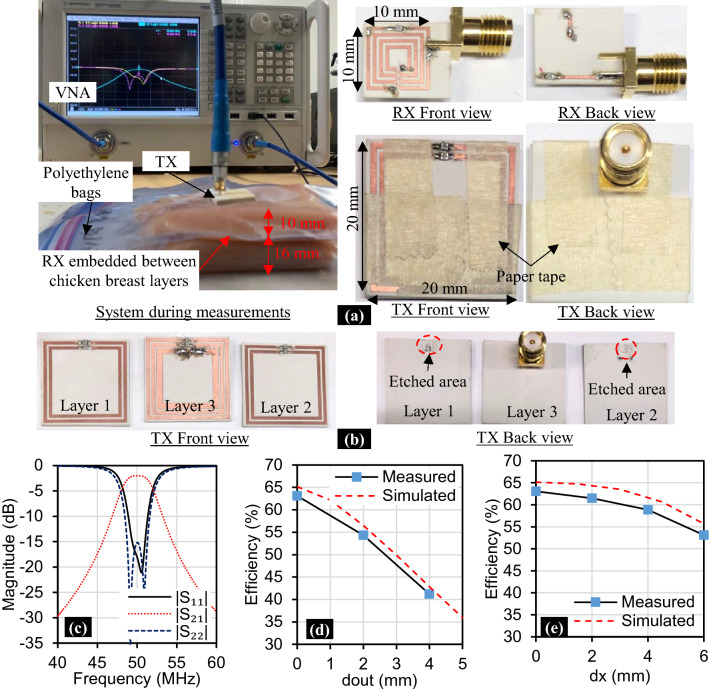
Fabrication and Measurements of the proposed Meta-TX WPT system. **(a)** System during measurement and fabricated RX/TX front and back views. **(b)** TX front and back views before assembly. **(c)** Measured S-parameters at perfect alignment. **(d)** Efficiency during TX separation misalignment. **(e)** Efficiency during lateral misalignment.

The Fabricated meta-TX and RX, as well as the experimental setup for measurements, are shown in Fig. 6a. The receiver is placed between two slices of chicken breast tissue with the dimensions shown in Fig. 6a. These tissue slices are placed inside polyethylene containers to ensure the necessary isolation layer between the copper traces of the meta-TX/tissue and RX/tissue as we have described in the simulations. Paper tape is used to fix the metamaterial layers together during the experiment. The view of the meta-TX layers is detailed in Fig. 6b. The substrates are etched with a layer sized 3 mm × 4 mm and a depth of 0.5 mm where the capacitors are bonded to ensure that there are no air gaps between the layers after assembly.

The measured transmission ($|{S}_{21}|$), TX reflection ($|{S}_{11}|$) and RX reflection ($|{S}_{22}|$) coefficients are shown in Fig. 6c, which confirm the operation at 50 MHz. Then, we compare the measured and simulated efficiencies during TX separation misalignment from the tissue surface and lateral misalignment as shown in Fig. 6d,e, respectively. The measured efficiency has good agreement with the simulated one during the misalignment. These results confirm the suitability of the proposed method to supply power to biomedical implants with high efficiency and rigidity to resonance change due to tissue proximity.

### Specific absorption rate and safety considerations

**Figure 7:**
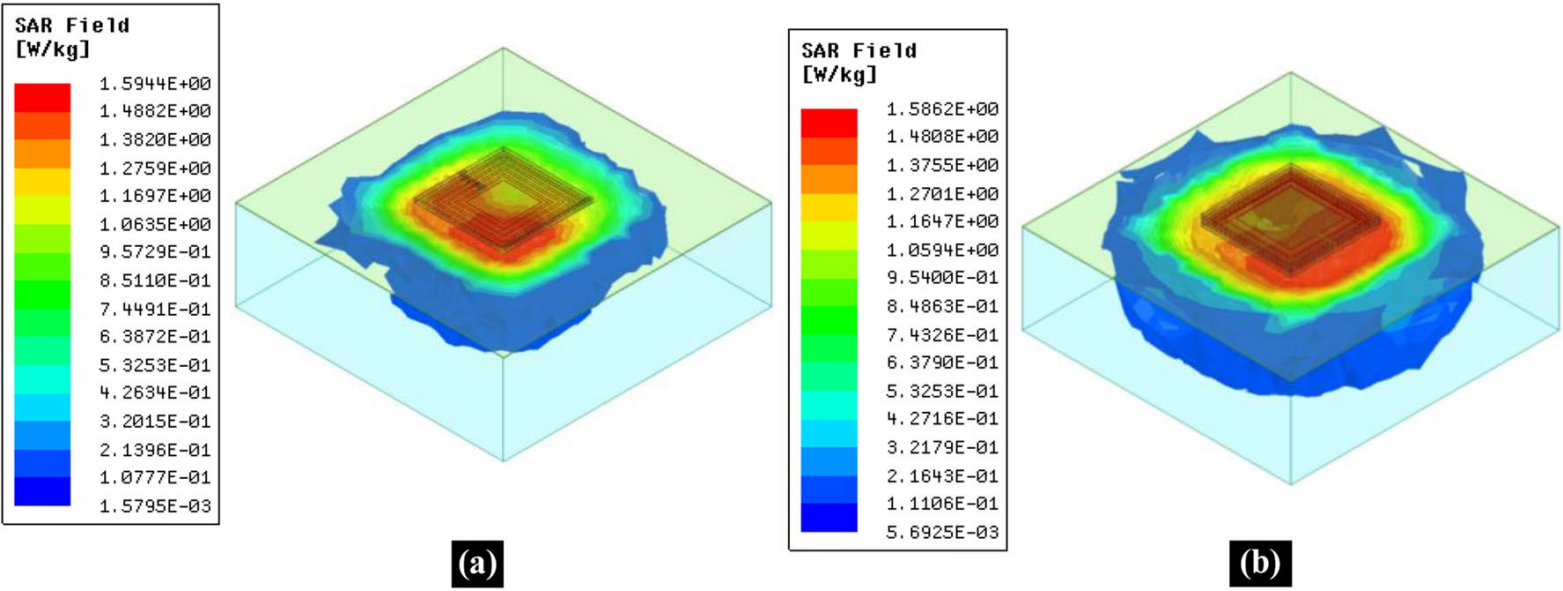
Three-dimensional view of EM Simulated 1-g average SAR distribution at 50 MHz **(a)** Conventional WPT system with 69 mW of input power. **(b)** Proposed metamaterial inspired WPT system with 168 mW of input power.

Ensuring the safety of the human body during exposure to EM waves is an indisputable fact. Specific absorption rate (SAR) is a measuring factor for EM wave absorption during exposure to these EM waves. SAR is calculated as^31^

9 $$SAR=\frac{{\sigma }_{tissue}}{{\rho }_{tissue}}{\left|E\right|}^{2}$$

where ${\sigma }_{tissue}$ is the conductivity of the tissue, ${\rho }_{tissue}$ is its mass density, and $E$ is the intensity of the induced electric field in the tissue due to exposure to these EM waves. A standard meter is the one-gram (1-g) averaged SAR as described in the IEEE standard C95.1-1999. This 1-g averaged SAR should not exceed 1.6 W/Kg to guarantee the safety of the human body. In Fig. 7a,b, we show the EM simulated 1-g average SAR at the 50 MHz operating frequency for the conventional WPT transfer system and the proposed stacked SRR metamaterial inspired WPT system, respectively. The maximum 1-g average SAR level in both cases is about 1.59 W/kg, i.e. it does not exceed the value defined by the IEEE standard C95.1-1999. However, these values have been achieved at different input power levels. The proposed metamaterial inspired WPT system can ensure the safety of the human tissue when the input power is 168 mW, which is about 2.5 times the input power in the case of the conventional system. The reason is that the proposed system has less radiation due to the minimization of the variance of the magnetic field in $\rho$-direction as described in the prior sections. Besides the improvement of misalignment performance, satisfying the 1-g averaged SAR limit^30^ while using 2.5 times higher input power than that in the conventional WPT system is another advantage of the proposed WPT system.

## Conclusion

We presented a metamaterial inspired geometry and used it as the transmitter of a WPT system to an embedded receiver in biological tissue. The unit cells of the proposed metamaterial-inspired geometry were coaxially aligned and stacked to shield each other from this tissue. The metamaterial inspired WPT system showed a negligible change in the resonance frequency when any airgap exists with the tissue, which was not the case in the conventional WPT system. Consequently, the proposed WPT system achieved a more stable efficiency during this kind of misalignment when compared to the conventional WPT system. Moreover, the proposed WPT system can operate with 2.5 times higher input power than that in the conventional system while fulfilling the limits imposed by IEEE standard C95.1-1999 for the 1-g averaged SAR. The proposed metamaterial inspired WPT system was fabricated and characterized during lateral and air-gap misalignment cases in chicken breast tissue. The measured and simulated results were in good agreement, which confirms the effectiveness of the proposed theory.

## Methods

### Electromagnetic simulations

We used ANSYS High-Frequency Structure Simulator (HFSS) for numerical electromagnetic simulations of the metamaterial as well as the wireless power transfer systems.

### Fabrication of samples

We fabricated the samples using the MITS FP-21 T Precision prototyping machine.

### Materials

We have used Rogers RO3003 (dielectric constant = 3, height = 0.762 mm, and copper thickness = 17 µm) during both of the electromagnetic simulations and the preparation of samples. Lumped capacitors have been implemented using the high-quality factor GJM series by Murata electronics to ensure no additional losses. In the case of large value capacitors, two parallel capacitors were used to realize the target value with an improved corresponding unloaded quality factor.

### Measurement setup

The measurement setup was composed mainly of a vector network analyzer, Keysight PNA series, part number: N5222A, and radio frequency cables. A chicken breast was used as the medium during the experiment. Chicken breast was bought from a supermarket (Grocery).
